# Combination of a Green and a Traditional Method for Estimating Relative and Absolute Ink Age: A Case Study of Ballpoint Pen Ink Dating in Vietnam

**DOI:** 10.1155/2021/8870541

**Published:** 2021-05-15

**Authors:** Anh Duc Hoang, Minh Binh Tu, Thi Thao Ta, Manh Hung Hoang

**Affiliations:** ^1^VNU University of Science, Vietnam National University–Hanoi, 19 Le Thanh Tong St., Hoan Kiem Dist., Hanoi 100000, Vietnam; ^2^Center for Consultancy, Civil Assessment–67 Khuat Duy Tien St., Thanh Xuan Dist., Hanoi 100000, Vietnam; ^3^Institute of Forensic Science-99 Nguyen Tuan St., Thanh Xuan Dist., Hanoi 100000, Vietnam

## Abstract

The dating of ink in questioned documents remains a significant challenge in forensic investigations in Vietnam and other countries. Many forensic examination methods have been usually applied to ensure the highest accuracy of the assessment results while maintaining high environment awareness. In this study, paper characteristics were physically tested to confirm source similarity, and the relative ink dating was established by high-performance thin-layer chromatography (HPTLC). Absolute ink dating by solvent and dye identification was performed by Raman spectrometry—a green technique, using a time-dependent degradation model for crystal violet and the comparison between 2-phenoxyethanol peak intensities. We found that the relative dating of the questioned document was 14 ± 3 months lesser than that of the reference samples, i.e., the absolute age of the questioned samples was estimated to be 24 ± 3 months. The combination of the conventional HPTLC method with the dynamic crystal violet degradation Raman model provides promising results for relative and absolute ink dating of ballpoint pens, which can be applied for documents written 1–15 years prior to the time of examination. The combination of the abovementioned methods demonstrated an acceptable error margin, affording highly practical applications in modern forensic science.

## 1. Introduction

Modern document examination often involves investigating the dating of written entries or signatures. This determination relies heavily on ink dating because document falsification usually occurs in the form of the addition or alteration of letters and figures. The importance of ink dating in forensic science began to attract attention only in the late 20th century when an ink reference collection was systematically created for forensic ink dating purposes [1]. Since then, analytical techniques have evolved, following the discoveries of different methods used in ink analysis for ink dating estimation. Researchers have been investigating these dating methods over the years due to the complex nature of ink, which can include components such as dyes, pigments, solvents, resins, lubricants, biocides, surfactants, corrosion inhibitors, sequestrants, shear-thinning agents, emulsifying agents, pH buffers, and other additives to control the ink characteristics (pH, viscosity, and polymerization) [2]. The increasing complexity of ink formulations can also be attributed to the following: (i) the continuous development of new ink products, with similar chemical properties, by manufacturers to reduce cost; (ii) the use of the same ink formulation for several different models by pen makers, or in some instances, the addition or replacement of particular components to improve the cost-effectiveness of the product; and (iii) the use of the same dye in different colors of ballpoint pen inks. For example, owing to the presence of methyl violet dyes in black and blue ballpoint pens [3], some models of a Vietnamese ballpoint pen brand displayed undifferentiable dye component profiles.

A typical ballpoint pen ink often contains approximately 50% solvents, 25% dyes, 25% resins, and 1% other additives [4]. Among the different solvent choices, 2-phenoxyethanol (2PE) is a main solvent that accounts for ∼80% of the solvent volume used in ink formulations. The estimation of the ink's age is still a significant challenge to forensic examiners because of the various decomposition mechanisms of the different components after ink is written on paper. The three suggested mechanisms that occur after ink deposition are solvent evaporation, resin solidification, and dye degradation. The rapid evaporation of solvents leads to the drying and diffusion of ink on paper, followed by color alteration due to dye or pigment degradation. Subsequently, solidification occurs because of the polymerization of resins [5, 6]. Among these, the process of dye degradation is the mechanism that is most resistant to environmental factors and occurs over very long periods, as suggested by several studies [4–8]. These temporal changes can be visualized by using high-performance thin-layer chromatography (HPTLC) methods, which separate dye components and develop them into colored spots on the thin-layer chromatography (TLC) plate. Although some studies have reported the use of HPTLC for the differentiation of ballpoint pen inks for forensic purposes in South East Asia and Australia-New Zealand regions [9–11], ink dating is still new to this geographical area, and to the best of our knowledge, studies on ink dating using HPTLC results have not been published yet.

In the initial studies on ink dating research [1, 12, 13], static approaches such as ink stroke measurement (size, color, hue, value, and chroma according to international notation and light reflectance) were used to differentiate inks. Other analytical methods such as ultraviolet-visible (UV-Vis) spectrometry [14], infrared luminescence spectrometry [15], near infrared-UV-Vis (NIR-UV-Vis) spectrometry [16], video spectral comparator [17], laser-induced fluorescence [13], and laser-induced infrared luminescence [18] were also applied to compare and identify inks. Ink analysis based on the extraction rate obtained using solvents with different polarities against a sufficiently expansive database of reference samples was also widely adopted as a relative ink dating method. It involved the sequential extraction of dyes to establish a temporal change in ink extractability [1, 5, 19–25]. This change, theoretically, is due to resin hardening, which results in the formation of a matrix that traps dye and solvent components inside, leading to a reduced extraction rate [26]. However, the reproducibility of this approach is still under debate [27, 28]. Therefore, it is often combined with other more advanced methods to improve the reliability and accuracy of the test results. Traditional TLC of low sensitivity and separation resolution has been used for this purpose until the advent of HPTLC, which along with the international standards used for inks, rendered HPTLC the most widely used method for comparing inks. Several researchers have successfully classified and identified ballpoint pens with a high degree of discriminating power using HPTLC methods [9, 10, 29–32]. Furthermore, ink analysis often involves the combination of HPTLC with other methods such as gas chromatography mass spectrometry (GC-MS) [33] and Fourier-transformed infrared spectrometry (FTIR) [34]. A few studies also combined Raman spectrometry with FTIR, with or without chemometrics, to classify and identify blue ballpoint pen inks [35, 36].

Raman spectrometry, which is considered a green method, is widely used for forensic examination not only due to its nondestructive and noninvasive effect on samples but also because it is fast, efficient, and waste-free [5, 37, 38]. Thus, it allows multiple measurements to be conducted without complex sample preparation, pretreatment, and harmful effects on the environment [5, 38]. Moreover, Raman spectrometry can be used to investigate sample sizes as small as <1 *µ*m with a low laser radiation power, thereby reducing the risk of sample degradation caused by laser irradiation [5, 38, 39]. Two recent studies on this method used the time-dependent curves of Raman peak intensity ratios for crystal violet (CV or basic violet 3, the most popular dye used in many black and blue ballpoint pen inks) and reported promising results with high dating accuracies, mitigation of concentration-related variability of peak intensities, and a wide practical estimation range of up to 15 years of ink age [4, 5].

The case study reported herein involves the investigation of a legal dispute in a court of law. The plaintiffs are suspected of attempting an unlawful seizure of the defendant's property, by writing new content on three blank sheets of paper presigned by the defendant. The questioned documents were sent for forensic examination to ascertain whether the age of contents written by the plaintiffs was similar to the age of the defendant's signatures. All the samples were written in black ballpoint pen inks (with visually detectable “striated” marks and ink deposits caused by ball tips, also confirmed by chemical spot test screening). Description of this preliminary step of writing instrument differentiation will not be included in this article.

According to a recent systematic review on ballpoint pen ink characterization and dating analysis, the majority of articles on black and blue ballpoint pen ink dating in the last decade have focused on differentiation and/or dating using analytical techniques that investigate a single parameter (2PE, CV, or resin polymerization) [40]. The relative dating methods are applied when the questioned documents contain two questionable inks which needed to be measured and compared, while absolute dating methods (also called dynamic dating) are used to investigate ink's components change over time [40]. In the present study, we aimed to concurrently investigate different parameters (CV, 2PE, and paper) using techniques such as the recently developed Raman spectrometry-based dynamic dating method [4] combined with HPTLC and paper characteristic measurements to estimate the relative and absolute questioned ink's age.

## 2. Materials and Methods

### 2.1. Materials and Chemicals

Paper attributes were measured using the following instruments: Mettler Toledo technical balance, Datacolor Elrepho 3000, Frank's Bekk smoothness tester, and Bendtsen air permeability tester. The measurements were obtained in accordance with the corresponding Vietnam national standards (TCVN) at the Research Institute of Pulp and Paper Industry Co., Ltd. The questioned documents were subjected to nondestructive examinations and tests for their physical properties. The parameters tested were mass, whiteness, opacity, smoothness, air permeability, and thickness.

For HPTLC, stock chemicals of butanol, isopropanol, ethanol, ethyl acetate, and acetone of analytical grade were purchased from Merck (Germany). CV from BDH (UK) and distilled water were used to prepare extraction and developing solvents. Extraction and TLC analysis on Merck silica gel 60 F_254_ plates were carried out in accordance with standards coded in ASTM E1789-04 (Standard Guide for Writing Ink Identification) [41] and ASTM E1422-05 (Standard Guide for Test Methods for Forensic Writing Ink Comparison) [42]. Brauband intraMark (Germany) capillary tubes were used for spotting 20 *µ*L of each extracted solution. Silica 60 F_254_ TLC plates (Merck, Germany) were used as the stationary phase, and the developing solvents used for TLC development were butanol/isopropanol/ethanol/water (5 : 3:2 : 1 v/v) (system A) and ethyl acetate/ethanol/water (26 : 13 : 11 v/v) (system B). The plates were developed in a CAMAG TLC Scanner IV system, and the retention factors (*R*_*f*_) and color intensity of the spots were assessed manually. *R*_*f*_ was calculated using the following formula:

(1) $$\begin{array}{c} R_{f}=\frac{\text{distance traveled by colorant}}{\text{distance traveled by developing solvents}}. \end{array}$$

### 2.2. Analytical Methods and Procedure

#### 2.2.1. High-Performance Thin-Layer Chromatography

Twenty microdots of the paper containing the ink were required for each HPTLC experiment. The paper extraction tool is a hole puncher with an inner diameter of 0.9 mm, handmade using a hypodermic 18G medical-grade needle tip made of stainless steel. The dots were taken from the center of the straight ink lines to ensure the whole width of the ink lines was removed at a homogenous length (which is the inner diameter of the puncher). The chosen ink lines were carefully selected to be of the same width and color density to minimize the variation of ink quantity.

The bevel head of the tip was sawn off across the diameter and filed to sharpen the contact edge. The twenty paper dots were then placed in test tubes and extracted with 20 *µ*l of ethanol/water/acetone 5 : 5:1 (v:v) and shaken for 1 minute. The whole extraction solutions were then spotted manually using capillary tubes on activated silica gel on the aluminum TLC plate (preheated at 100°C for 20 min for activation). The developing solvents were allowed to saturate in developing chambers for 30 min before submerging the spotted plates for conventional vertical development. The plates were air-dried, and the *R*_*f*_ and color intensities of spots were manually recorded.

The repeatability of this method was tested by spotting five spots of a ballpoint pen ink on the same plate,conducted by the same technician. To determine the reproducibility, different technicians prepared and developed a similar plate on three consecutive days. The relative standard deviations (RSDs) of the repeatability and reproducibility were within 5%.

#### 2.2.2. Raman Spectrometry

Raman spectra were acquired with a Nicolet 6700 Raman spectrometer with an NXR FT-Raman Module of Thermo Finnigan. The settings used were as follows: number of sample scans, 160; detector, InGaAs; beamsplitter, CaF_2_; resolution, 8.000; sample gain, 2.0; mirror velocity, 0.3165; aperture, 100.00; laser power, 0.3 W; and laser wavelength, 1064 nm.

Raman spectra were obtained at low laser power to avoid burning the samples. The fluorescence at a laser wavelength of 1064 nm is insignificant. The spectra for the Raman shifts were recorded in the 300–3700 cm^−1^ range, although the interested range will mainly be between 400 and 1700 cm^−1^, which is the fingerprint area (i.e., skeletal vibrations of a compound). Baseline correction (1 iteration) and smoothing (3 iterations) were performed for processing the Raman spectra.

The repeatability and intraday reproducibility were determined by measuring the same spot without removing/replacing the sample or changing the parameters. Seven measurements on the same day for repeatability resulted in RSD ≤ 0.87%. Five measurements on five different days for reproducibility resulted in RSD ≤ 1.41%.

Peak intensities of CV at 729 cm^−1^ and 1580 cm^−1^ were recorded, and peak intensity ratios between these (PIR_729/1580_) were calculated and plotted versus absolute ink age at the time of examination (months), following Gorshkova et al. CV time-dependent degradation curve [4].

### 2.3. Sample and Preparation

**Figure 1:**
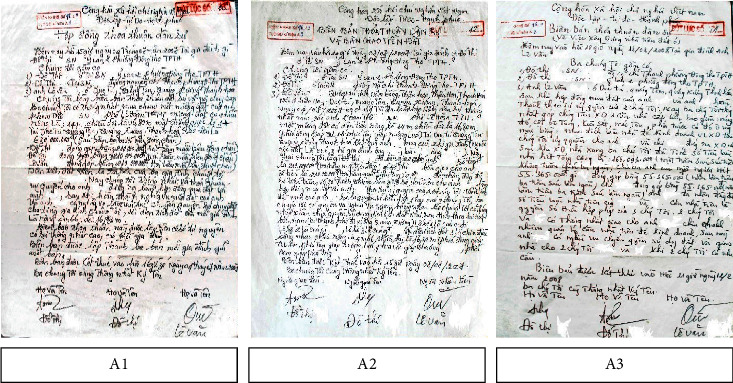
Questioned documents (A1, A2, and A3) (deidentified with white markers).

All samples were obtained from a court, and the papers under examination were coded as A1, A2, and A3 (Figure 1). In particular, the content parts of three papers (excluding the signatures), coded A1.1, A2.1, and A3.1, were all written by the plaintiffs, and the handwritings were previously assessed to be genuine by provincial examiners. The reference samples coded A1.2, A2.2, and A3.2 (39 months old) were signatures and full names of the defendant at the bottom right of A1, A2, and A3, respectively. The other reference samples M1, M2, and M3 were 50 months old, and these were the land purchase contracts and receipts between the defendant and the previous landowner, certified by local authorities. The argument that remained was that the plaintiff stated that the contents had been written on the same date that the defendant signed on each paper, whereas the defendant claimed that he had presigned on empty paper sheets for business purposes and he was unaware of the contents added during the business fallout.

Ballpoint pen writing can be differentiated from other type of writing instruments quite easily by a few simple methods: (i) visual inspection: ballpoint pen writing leaves unique features such as “striated” lines and ink deposits caused by uneven distribution of ink on the ball tip, which does not occur with any other pen tip; (ii) chemical spot tests: ballpoint pen inks are mostly oil-based therefore often easily dissolved in glycol-based solvents (e.g., n-butanol), while most gel inks cannot be dissolved by glycol-based solvents (except some Pentel gel pens). Description of this preliminary step was not included in this article, but in fact a must-have basic step in forensic document analysis.

Sample preparation was not required for the measurements of paper characteristics, and Raman spectrometry, a nondestructive technique, was performed directly on the questioned documents. Since HPTLC is a minimally destructive technique, microdots of the paper containing the questioned ink were removed from the samples and transferred to test tubes for extraction of the ink components.

## 3. Results and Discussion

### 3.1. Analysis of Paper Characteristics

The characteristics of the paper measured (Table 1) were mass, whiteness, opacity, Bekk smoothness, air permeability, and thickness. The RSDs of all measurement results ranged from 0.25% to 4.67% (less than 5%); therefore, 3 paper sheets were likely sourced from the same batch (or the same quire of paper) of the same paper mill.

These properties are affected by environmental factors such as temperature, humidity, and light, as well as aging or conservation treatments. Therefore, a comparison of these characteristics can shed light on the similarity of the paper source and aging status [43]. For example, if these papers were from different sources, then it is unlikely that they were written on at the same time as claimed by the defendant and vice versa (despite the similar appearances as seen in Figure 1). This finding also suggested that the 3 papers were likely kept under similar storage conditions. To the best of our knowledge, studies for assessing the paper substrate with or without a combination of other methods for ink dating purposes are sparse.

### 3.2. Separation of Dye Components and Relative Dating Estimation by HPTLC

Methanol was selected as the solvent for extracting the ballpoint pen ink on paper in many articles. Despite its high toxicity, several authors have employed it because of its high extraction efficiency [33, 34, 44–49]. In this case study, extraction with ethanol/water/acetone (5 : 5:1 v/v) showed good extraction efficiency, and most importantly, this extracting solvent was less toxic to lab technicians compared to methanol, thus improving the safety profile of the experiment.

**Figure 2:**
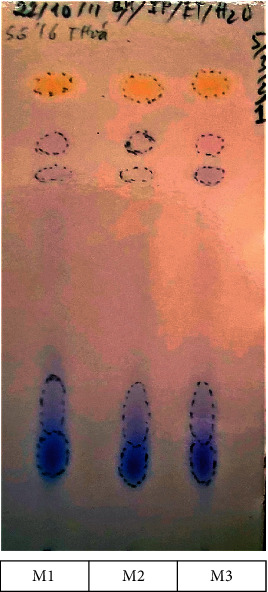
HPTLC of reference samples M1, M2, and M3 (50-month-old) developed in system A—butanol/isopropanol/ethanol/water (5 : 3:2 : 1 v/v).

**Figure 3:**
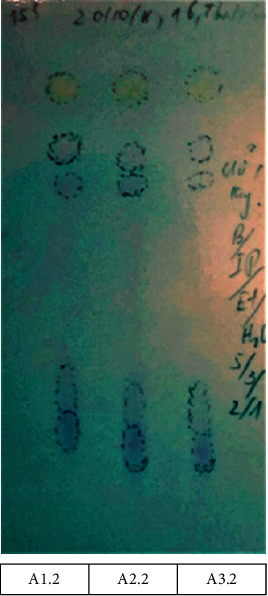
HPTLC of reference samples A1.2, A2.2, and A3.2 (39-month-old) developed in system A—butanol/isopropanol/ethanol/water (5 : 3:2 : 1 v/v).

**Figure 4:**
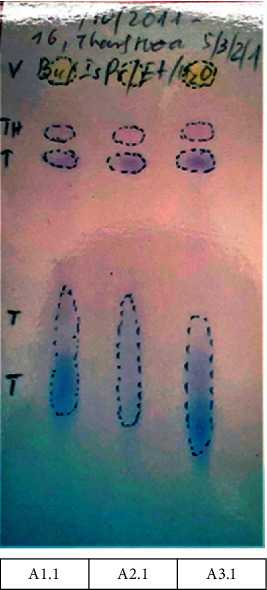
HPTLC of questioned samples A1.1, A2.1, and A3.1 developed in system A—butanol/isopropanol/ethanol/water (5 : 3:2 : 1 v/v).

Developing systems A and B were chosen based on Roux et al. ballpoint pen differentiation study with minor modification in the ratio of system A [11]. In system A (Figures 2–4), all inks showed a similar number and color of spots. Therefore, they contained similar ink components; hence, only one sample from each group was selected as a representative for further analysis.  Figure 2 shows the reference samples M1, M2, and M3 (50 months old) with similar *R*_*f*_ and color intensities of the spots in system A. The reference samples A1.2, A2.2, and A3.2 (39 months old) showed similar *R*_*f*_ and color intensities of the spots in system A (higher *R*_*f*_ and lighter color intensity compared with that of M1, M2, and M3) (Figure 3). The questioned samples A1.1, A2.1, and A3.1 also displayed a similar *R*_*f*_ and color intensity as those of the spots in Figure 4. Therefore, they were likely to have been written around the same time. Furthermore, they had the highest *R*_*f*_ and the lightest color compared with all reference samples, indicating that the questioned samples were written on dates after all the reference samples.

**Figure 5:**
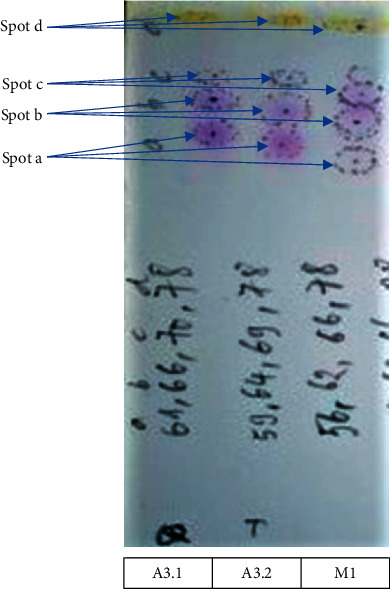
HPTLC of A3.1, A3.2 (39-month-old), and M1 (50-month-old) developed in system B—ethyl acetate/ethanol/water (26 : 13 : 11 v/v).

The plates in system B also displayed similar ink components as those of the three representative samples A3.1, A3.2, and M1, with an increase in the *R*_*f*_ and color intensity from M1 (50-month) < A3.2 (39-month) < A3.1 (Figure 5), supporting the results obtained from system A. The three violet spots on the plate correspond to CV, pentamethyl pararosaniline chloride, and tetramethyl pararosaniline chloride; the latter two compounds are the photodegradation products of CV [50, 51]. The measurement results are summarized in Table 2. Owing to the ink resin hardening, which reduces the extraction rate of the dye, the TLC spots of the older sample M1 showed a lower *R*_*f*_ and lower color intensity than those of the newer reference sample A3.2. Therefore, the results from system B suggested that A3.1 was written more recently than A3.2, i.e., the relative dating of A3.1 is less than 39 months old.

### 3.3. Absolute Dynamic Dating Estimation by Raman Spectrometry

Based on a newly developed model of ink aging estimation, which claims a high accuracy of over 85% of tested samples [4], peaks at 729 cm^−1^ and 1587 cm^−1^ were selected to investigate changes due to varying susceptibility to the aging parameters of CV, a triarylmethane dye that exists in many black and blue ballpoint pen inks [4, 5]. The peak at 729 cm^−1^ represents vibrations of peripheral C-N bonds, which are most susceptible to changes over time. The peak at 1580 cm^−1^ represents the vibrations of the most stable bonds in the CV molecule, which in this case are the C-C bonds in the chromophore aromatic rings.

The ratio of peak intensity was used for comparison instead of absolute intensity, thus eliminating the concentration dependency at the focal spot of the laser beam, which causes inconsistencies in the Raman intensities measured at different spots on the same ink line. The degradation process of CV is divided into three monotonic periods, as proposed by Grechukha et al. [5]. Using this parabolic estimation to plot the position of reference samples, the dating of the questioned samples can be estimated with high accuracy.

Dhakal et al. [52] reported the major components identified in black ballpoint pen inks by paper spray–mass spectrometry methods that identified two major solvents and six major dyes. In our case, we were able to detect two dyes and one solvent which have strong similarity to the major Raman peaks of the three samples M1, A3.2, and A3.1.

For instance, a strong similarity between the intense peaks and the literature CV data at 524 cm^−1^, 724 cm^−1^, 915 cm^−1^, 1178 cm^−1^, 1369 cm^−1^, 1536 cm^−1^, and 1591 cm^−1^ can be observed. Furthermore, less intense CV peaks were also observed at 1300 cm^−1^, 1447 cm^−1^, and 1622 cm^−1^, which may be attributed to the interactions with other ink components. The reference Raman shifts of the CV [4] and their corresponding type of vibrations are described in Table 3. This finding identified the purple spots developed on the HPTLC as CV and the two other degradation products, as discussed above. Yellow spots were observed on the HPTLC plate suggesting it to be metanil yellow (MY) due to the intense peaks that match the reference Raman fingerprint of solid MY at 995 cm^−1^,1147 cm^−1^, and 1188 cm^−1^ [52] which overlap with the CV peaks.

In the previous TLC section, although the yellow spots had clear intensity differences, their *R*_*f*_ values were indistinguishable; therefore, the CV component alone was used to estimate the dating of the questioned samples owing to the considerable differences in both *R*_*f*_ and color intensity.

**Figure 6:**
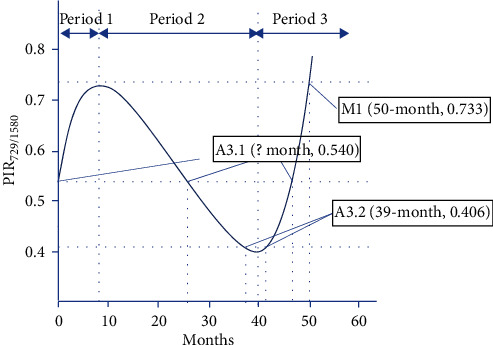
Estimation of ink dating by the time dependency model of CV with modified period 3.

Estimation of the ink date was based on the time-dependent curve proposed by Gorshkova et al. [4]. The peak intensity ratios (PIRs) (726 cm^−1^/1586 cm^−1^) measured for the three samples were PIR_M1_ = 0.733, PIR_A3.1_ = 0.540, and PIR_A3.2_ = 0.406 (plotting as shown in Figure 6). The time of sample acquisition was recorded 50 months from the dating of M1, which led to an inaccurate estimate if plotted following the original curve, likely due to other various components of this particular type of ballpoint ink and/or different storing conditions, that caused a more rapid cleavage of the C-phenyl bonds of the central chromophore. Therefore, the slope of period 3 needs to be adjusted accordingly to correctly reflect this case-by-case variance. The polynomial curve was reconstructed from 3 anchor points: peak point at 8-month with PIR = 0.725, bottom point at 40-month with PIR = 0.4 (both theoretical by Gorshkova), and M1 at 50-month with PIR = 0.733 (practical value from Raman spectra measurements), resulting in a steeper slope for period 3.

The absolute dating of A3.2 of 39 months while plotting the Raman peak intensities showed 2 variables at 37.5 months and 42 months. These data points reflect the low tolerance of estimation on period 2 of the curve (at *a* ± 3-month tolerance).

The plot of A3.1 indicated three points on the curve, which matched the peak intensity ratio of 0.540, and represent the 0th month, 25th month, and 46th month from the acquisition (the 46th month was estimated from data points of two reference samples M1 and A3.2). As suggested by the TLC results presented, A3.1 had an absolute dating of less than 39 months; therefore, 25 months was the only logical estimation (0 month did not contradict TLC findings; however, it was impossible since the questioned documents had been stored at the court for 5 months before examination), i.e., the age order reflecting the TLC result: A3.1 (25-month) < A3.2 (39-month) < M1 (50-month). Since this dynamic aging model was proposed in 2017, to the best of our knowledge, there was no report of its application in the casework.

On another note, the solvent identified in the spectra of the three ballpoint pen ink samples was 2PE. Peaks matching 2PE-containing triarylmethane dye reference were seen at 570 cm^−1^, 729 cm^−1^, 765 cm^−1^, 915 cm^−1^, 1177 cm^−1^, 1308 cm^−1^, 1450 cm^−1^, and 1587 cm^−1^ in all three samples with the following order of intensity: *I*_A3.1_ > *I*_A3.2_ (39 months)> *I*_M1_ (50 months). Since the absolute Raman peak intensities are considered directly proportional to the concentration of the analyzed materials [52] when all other conditions are kept constant, including the paper substrates which were assessed to be from the same batch previously, this result can be interpreted as the concentration of 2PE remaining in the questioned sample A3.1 being higher than that in the reference sample A3.2 at the relative age of 39 months, which, in turn, is higher than that of the reference sample M1 at the relative age of 50 months.

The concentration of the volatile components, i.e., solvents used in the ink formulation, rapidly decreases because of evaporation when they are first deposited on paper. However, after a certain period, this process considerably slows down, reaching a near-plateau-shaped curve [4, 53]. This period is usually short under natural conditions, taking from several days to 2 weeks for 90% of the 2PE solvent to evaporate. Bügler et al. [54] reported that with a thermal desorption-based GC-MS method, 2PE-based dating could only be estimated for up to several months, while Brazeau and Gaudreau [53] were able to detect 2PE in samples that were up to nearly 2 years old. Regarding our case study, the concentrations of 2PE indicated by the aforementioned peak intensities were less significant compared with that of CV or MY, indicating that these samples had all aged past the rapid evaporation period of the ink solvent. The differences observed from the peak intensities of 2 PE peaks were detectable, which may be due to the contents of the questioned document written significantly later (i.e., newer) than the signature of the defendant on the same paper, which has already aged into the near-plateau section of the 2PE concentration-vs-time curve proposed by Gorshkova et al. [4]. This possibility is supported by other findings in this experiment.

## 4. Conclusions

This study successfully employed green methods (Raman and paper characteristics measurement) with the traditional HPTLC method for ink analysis to estimate relative and absolute ink dating. Our dating results were based on new approaches such as paper characteristic analysis to confirm batch similarity and storage conditions and interpretation of the HPTLC result by considering both color intensity and *R*_*f*_ values to establish a relative dating sequence. Most importantly, the use of Raman peak intensity ratios enabled the dating of the CV-containing ink with a practical error margin of ±3 months. The accuracy of the methodology could be improved by using sufficiently large databases to mitigate the possibilities for the erroneous adjustment of the time-dependent curve and the limited availability of reference samples. For example, the time-dependant curve could have been reconstructed more accurately with added number of reference samples instead of only 2 available reference samples in this practice case. Furthermore, the application of other modern quantitative green analytical methods can further minimize impacts on the environment. In summary, with a more in-depth quantification of the studied parameters, our results will contribute to the development of a promising multidisciplinary approach for reliable ink dating in practical forensic science.

## Figures and Tables

**Table 1:** Physical characteristics of papers.

		Standards (TCVN)	Questioned documents				RSD (%)
			A1		A2	A3	
1	Mass (g m^−2^)	1270 : 2008	59.5		61.1	60.1	≤1.44
2	Whiteness (ISO, %)	1865-1 : 2011	91.1		90.8	90.7	≤0.25
3	Opacity (%)	6729-2008	86.2		88.6	88.4	≤1.74
4	Bekk smoothness (second)	6727 : 2007	Side 1	59.3	62.0	56.5	≤4.67
			Side 2	27.5	28.8	27.0	≤3.72
5	Air permeability (ml min^−1^)	6891 : 2001	1385		1392	1356	≤1.57
6	Thickness (mm)	N/A	0.041		0.039	0.040	≤2.5

**Table 2:** HPTLC *R*_*f*_ values of A3.1, A3.2, and M1 samples developed in solvent system B (from left to right in Figure 5).

*N *_o_	Spot	*R *_*f*_ of A3.1	*R *_*f*_ of A3.2 (39 months)	*R *_*f*_ of M1 (50 months)	Color under visible light	Remark
1	a	0.74	0.72	0.71	Violet	Color intensity and *R*_*f*_ value: A3.1 > *A*3.2 > M1
2	b	0.80	0.78	0.76	Violet	
3	c	0.85	0.84	0.83	Violet	
4	d	0.95	0.94	0.93	Yellow	

**Table 3:** Peak wavelength number (cm^−1^) and reference data for reference dyes and samples M1, A3.2, and A3.1.

M1 (50 months)	A3.2 (39 months)	A3.1 (date)	Reference data [6]	Type of vibrations
525	**525**	**525**	**524 (CV)**	***δ* (CNC)**
+	**+**	**+**	**558 (CV)**	***γ* (CCC)/*δ* (CNC)/*δ* (CC **_**center**_ ** C)**
726	**724**	**726**	**724 (CV)**	***ν* (CN)**
912	**916**	**912**	**915 (CV)**	***ν* (CC) **_**ring**_
*997*	*993*	*997*	*995 (MY)*	
*1137*	*1141*	*1141*	*1147 (MY)*	
1184	**1180**	**1180**	**1178 (CV)**	***ν***_**s**_ ** (CC **_**center**_ ** C)/*δ* (CCC) **_**breathing**_ ** /*δ***_**r**_ ** (CH **_**3**_ ** )**
*1188*	*1188*	*1188*	*1188 (MY)*	
1300	**1295**	**1306**	**1300 (CV)**	***ν***_**as**_ ** (CC **_**center**_ ** C)/*δ* (CCC) **_**ring**_ ** /*δ* (CH)**
1361	**1365**	**1365**	**1369 (CV)**	***ν* (CC **_**center**_ ** C)**
1430	**1436**	**1428**	**1447 (CV)**	***δ***_**as**_ ** (CH **_**3**_ ** )**
1539	**1540**	**1534**	**1536 (CV)**	***ν* (C **_**ring**_ ** N)/*δ***_**s**_ ** (CH **_**3**_ ** )**
1586	**1582**	**1586**	**1591 (CV)**	***ν* (C-C) **_**ring**_
+	**1622**	**1622**	**1622 (CV)**	***ν* (C-C) **_**ring**_

*v*, stretching vibrations; *δ*, planar deformation vibrations; *s*, symmetric; “+,” small peak observed; as, asymmetric. Values in bold indicate the matching peaks of crystal violet (CV). Values in italics indicate the matching peaks of metanil yellow (MY).

## Data Availability

The data used to support the findings of this study are available from the corresponding author upon request.
